# Identification of Lactate as a Cardiac Protectant by Inhibiting Inflammation and Cardiac Hypertrophy Using a Zebrafish Acute Heart Failure Model

**DOI:** 10.3390/ph14030261

**Published:** 2021-03-15

**Authors:** Elijah R. Haege, Hui-Chi Huang, Cheng-chen Huang

**Affiliations:** 1 Biology Department, University of Wisconsin-River Falls, River Falls, WI 54022, USA; ehaege123@gmail.com; 2 Department of Chinese Pharmaceutical Sciences and Chines Medicine Resources, China Medical University, Taichung 40402, Taiwan; 3 Master Program for Food and Drug Safety, China Medical University, Taichung 40402, Taiwan

**Keywords:** acute heart failure, zebrafish, lactate, inflammation, cardiac hypertrophy

## Abstract

Acute heart failure (AHF) commonly arises from decompensated chronic heart failure or sudden structural and functional breakdown causing a decrease in cardiac contractility and consequently fluid accumulation and systemic congestion. Current treatment for AHF aims at reducing fluid overload and improving hemodynamic which results in quick symptom relief but still poor prognostic outcome. This study utilizes a zebrafish AHF model induced by aristolochic acid (AA) to look for natural products that could attenuate the progression of AHF. The project started off by testing nearly seventy herbal crude extracts. Two of the positive extracts were from Chinese water chestnuts and are further studied in this report. After several rounds of chromatographical chemical fractionation and biological tests, a near pure fraction, named A2-4-2-4, with several hydrophilic compounds was found to attenuate the AA-induced AHF. A2-4-2-4 appeared to inhibit inflammation and cardiac hypertrophy by reducing MAPK signaling activity. Chemical analyses revealed that the major compound in A2-4-2-4 is actually lactate. Pure sodium lactate showed attenuation of the AA-induced AHF and inflammation and cardiac hypertrophy suppression as well, suggesting that the AHF attenuation ability in A2-4-2-4 is attributable to lactate. Our studies identify lactate as a cardiac protectant and a new therapeutic agent for AHF.

## 1. Introduction

Acute heart failure (AHF) is defined as new or worsening of symptoms and signs of heart failure. There are two types of AHF, de novo AHF and acute decompensated HF (ADHF). De novo AHF typically occurs in patients who have no previous history of heart failure but develop acute or chronic cardiac pathologies, such as cardiac ischemia, myocardial infarction, inflammatory insults, toxin insults, such as anthracycline [1,2] or chemotherapeutic agent [3], and others. The underlying cardiac pathologies first cause decreased contractility in myocardium followed by activation of several compensatory pathways as in chronic heart failure to briefly bring up the cardiac output but eventually lead to fluid accumulation, systemic congestion and organ dysfunction [4]. ADHF, which accounts for the large majority of AHF cases, occurs in patients with previously diagnosed chronic HF when the abovementioned compensatory physiological systems force the heart to exceed its limit. 

As the common presentation of AHF is the accumulation of fluid, current treatment for AHF is aimed at supporting hemodynamic compromise, which includes loop diuretics, vasodilators and positive inotropes. Although many current treatments show quick relief of symptoms, they fail to significantly reduce the mortality of AHF within six months [5]. One reason could be that patients with ADHF often have co-morbidities, such as atrial fibrillation, valvular heart disease and others [6]. Similarly, de novo AHF could be complicated by a number of conditions, not exclusively with cardiac contractility failure [7]. The tremendous heterogeneity of the AHF pathophysiology is likely the reason why AHF patients have very different responses to the current treatment and poor prognostic outcome, with 90-day readmission rates and 1-yr mortality reaching 10–30% [8,9]. Therefore, it is recommended that specific treatments for the underlying cardiac disease and the precipitating factors should be implemented during hospitalization [4]. 

Inflammation is a defense response mediated by immune cells to remove pathogens, toxic compounds, and damaged cells [10,11]. While inflammation is vital for host defense and tissue repair, prolonged inflammation can cause further tissue damage and chronic inflammatory diseases [12,13]. For example, myocardial infarction caused by ischemia typically triggers inflammation to restore blood flow, which was found to cause further tissue destruction as a result of neutrophil infiltration and enhanced production of reactive oxygen species [14]. Thus, inhibition of inflammation has been believed and shown to be one of the most effective ways to protect injured organs and tissues [15,16]. Not surprisingly, the inflammation system has been found to associate with and become another risk and predictive factor for heart failure progression [17]. But due to the complex and still poorly understood physiological interplay preceded or triggered by heart failure, clinical and basic studies attempting to treat heart failure through inhibiting inflammation so far have shown mixed results.

Lactate, which was long believed to be a metabolic waste from glycolysis, was recently found to be a signaling molecule capable of modulating inflammation [18]. In the past two decades, studies have shown the beneficial role of lactate in several pathophysiological processes. First, lactate was found to accelerate wound healing and prevent ischemic skeletal muscle atrophy [19]. Lactate was shown to reduce toxin-induced liver and pancreatic injury by inhibiting inflammation [16]. In a study on ischemic brain injury, a high concentration (20 mM) of lactate contributed to neuroprotection while, interestingly, a low concentration (1–3 mM) of lactate caused neuronal injury [20], which seems inconsistent with the opposite effects of different levels of anti-inflammation on tissues and cells reported by other studies.

Many animal models have been established to study heart failure [21]. While these models are excellent tools for heart failure studies, few of them are ideal for drug discovery. For example, the murine models are expensive and time- and labor-intensive. Zebrafish embryos have shown many advantages for human disease studies and drug discovery [22]. We previously reported a zebrafish AHF model using aristolochic acid (AA) that exhibits phenotypes closely resembling those of human heart failure, including cardiac hypertrophy, severed cardiac fibers, loss of endocardium, and gradual weakening and subsequent cessation of cardiac contractility [23]. Two of the current drugs for human heart failure, metoprolol (a β-blocker) and captopril (an angiotensin converting enzyme inhibitor, or ACE-I), also showed a beneficial effect on AA-induced AHF. Furthermore, the anti-inflammation drug NS398, which selectively inhibits COX-2, could moderately attenuate the AHF in our model as well. Thus, our toxin-induced heart failure model presents a promising avenue to drug discovery for AHF [23].

Here we report the results from our continuous attempt of using our AHF zebrafish model to look for natural product that is beneficial to AHF. We first carried out a random test on near seventy herbal crude extracts. Two of the nine positive crude extracts, both from Chinese water chestnuts, were selected for a series of bioassay-guided isolation leading to the identification of the final positive fraction, named A2-4-2-4. Chemical analyses revealed that the major compound in A2-4-2-4 is lactate. We went on to show that pure sodium lactate could phenocopy A2-4-2-4, strongly suggesting that the heart failure attenuation activity in A2-4-2-4 is likely due to lactate. Our results reveal that lactate could be a cardiac protectant for toxin-induced and possibly other forms of AHF. However, the translation of our results to a clinical scenario requires additional experiments in animal model and human subjects.

## 2. Results

### 2.1. Chemical Extraction

The project designed to use our AA-induced zebrafish AHF model to look for potential AHF drugs from natural sources. We first collected sixty nine crude extracts containing compounds from various Chinese herbs which were extracted with different solvents, including water, methanol, ethyl acetate, butanol, etc. (the list of herbs could be found in Figure S1). The crude extracts were then condensed and re-dissolved in DMSO to make an arbitrary 100 mM concentration (see Section 4.3 for Chemical Preparation) and were then tested with the zebrafish AHF model. We performed four trials on all the extracts and identified nine herbal extracts that showed AHF attenuation in one or more trials (Figure S1, detailed AHF attenuation assessment could be found in Section 4.3). Four of them (highlighted in yellow) showed AHF attenuation consistently in all four trials and likely contain compounds that are beneficial to AHF. Two of them, named C2 and C3, both contain compounds extracted from cooked fruit of Chinese water chestnuts *(Trapa taiwanensis Nakai)*, with C2 by 50% ethanol and C3 by water.

### 2.2. Bioassay-Guided Chemical Fractionation

**Figure 1:**
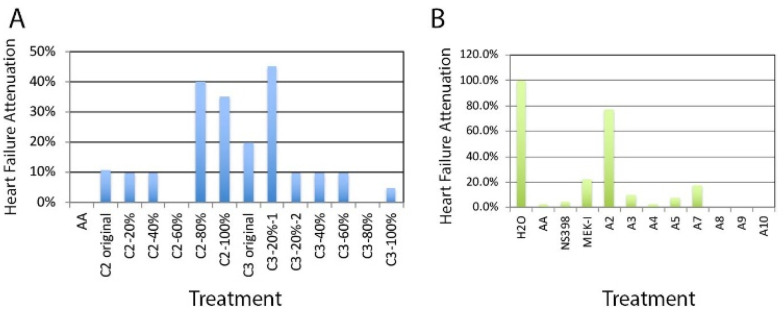
AHF attenuation by *Trapa taiwanensis Nakai* compounds. Two crude extracts from Chinese water chestnuts (*Trapa taiwanensis Nakai)* were generated using different solvents, 50% ethanol for C2 and water for C3. Initial tests using our zebrafish model showed AHF attenuation activity. Both C2 and C3 extracts were proceeded with medium pressure column chromatography (MPLC) to generate fractions eluted out with methanol gradient, 20%, 40%, 60%, 80% and 100%. Due to the large volume, C3-20% was further divided into two fractions, C3-20%-1 and C3-20%-2. (**A**) C3-20%-1 showed greater than 40% AHF attenuation. Although C2-80% and C2-100% also showed attenuation in this representative experiment, they failed to show the same activity in the later experiments. (**B**) The compounds in C3-20%-1 were further separated into ten fractions, named A1 to A10. A2 repeatedly showed significant AHF attenuation activity. A1 and A6 were not tested due to a too-little yield of compounds.

By employing Dianion column chromatography, compounds in C2 and C3 crude extracts were fractionated using methanol/water (MeOH/H_2_O) gradient (20/80 to 100/0, *v*/*v*) and tested with the zebrafish AHF model. Fractions C2-80% (methanol), C2-100%, and C3-20% appeared positive while other fractions showed little or no attenuation (Figure 1A). Fraction C3-20% was next fractionated using C_18_ MPLC (medium pressure column chromatography) using a methanol gradient to yield ten fractions, A1-A10. We found that only the A2 fraction exhibited an AHF attenuation ability (Figure 1B).

**Figure 2:**
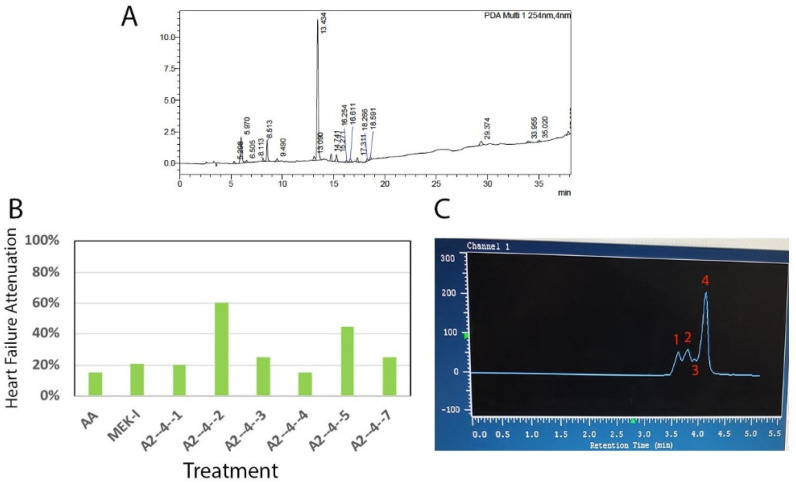
A2-4-2-4 purification. (**A**) HPLC analysis showed many compounds in the A2 fraction. (**B**) The A2-4 fraction was further separated into seven fractions, A2-4-1 to A2-4-7. Among them, A2-4-2 showed the highest AHF attenuation activity. (**C**) A representative UV absorbance graph during the preparative HPLC for A2-4-2 revealed that the A2-4-2 fraction contains four UV absorbance peaks which are significantly overlapped. Although extreme care was taken to separate the compounds into the final four fractions, named A2-4-2-1 to A2-4-2-4, the NMR analysis eventually showed impurities in A2-4-2-4 (see Figure 3).

**Figure 3:**
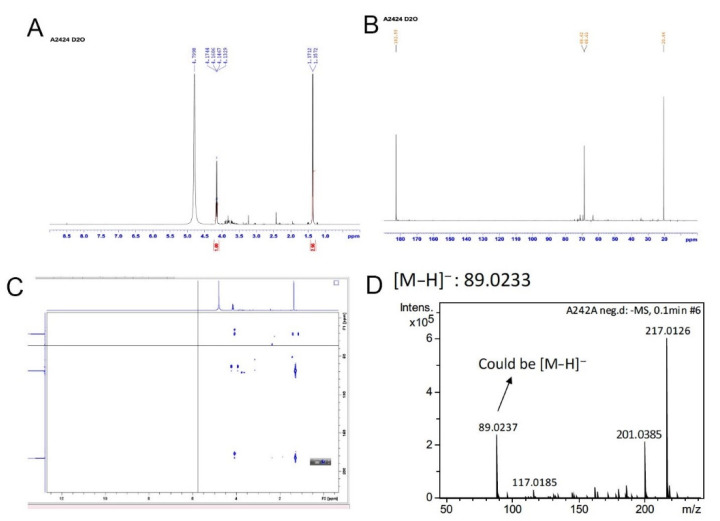
Chemical analyses for A2-4-2-4. A2-4-2-4 was analyzed using NMR and ESIMS. (**A**) The ^1^H-NMR graph indicates a methyl group (δ_H_ 1.3, d, *J* = 7.0 Hz) and a methane group (δ_H_ 4.16, q, *J* = 7.0 Hz) in A2-4-2-4. (**B**) The ^13^C-NMR indicates a carboxyl group (δ_C_ 182.5), a hydroxyl group (δ_H_ 68.6), and a methyl group (δ_H_ 20.4) in the major compound in A2-4-2-4. (**C**) The 2-dimensional NMR, using heteronuclear multiple bond correlation, further confirmed that the major compound in A2-4-2-4 is lactate. (**D**) ESIMS analysis revealed that the molecular weight of the major compound in A2-4-2-4 is 89.0233 (C_3_H_6_O_3_).

The chemical profile of A2 fraction revealed by HPLC showed a large number of compounds in it (Figure 2A). Next, the phenotype-guided procedure was repeated several times until the final partially purified fraction named A2-4-2-4 was identified (detailed in the Materials and Methods section and a representative piece of data is shown in Figure 2B). A2-4-2-4 is the fourth peak in the HPLC fractionation attempt for the parent A2-4-2 fraction using 0.5% methanol (Figure 2C). A2-4-2-4 was then characterized by several chemical methods. First, from ^1^H-NMR (nuclear magnetic resonance) and ^13^C-NMR, we found that A2-4-2-4 is actually a mixture of possibly three compounds (Figure 3A,B). The major signals on the NMR graphs indicate a compound with three carbons in a methyl, a methane, and a carboxyl group. Two-dimensional NMR and ESIMS (electrospray ionization mass spectrometry) analyses (Figure 3C,D) further confirmed the chemical groups and molecular weight, respectively, of the major compound in A2-4-2-4 to be lactate. Because the compounds in A2-4-2-4 are all very hydrophilic and eluted together with 0.5% methanol, it seems impossible to further separate them into pure compounds.

### 2.3. Comparisons between A2-4-2-4 and Other Drugs

**Figure 4:**
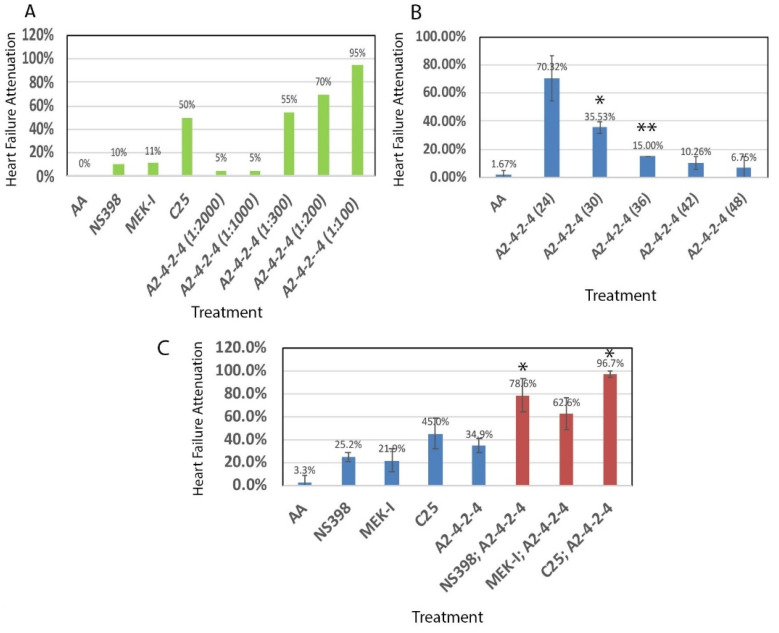
A2-4-2-4 pharmacodynamics. (**A**) A2-4-2-4 showed the heart failure attenuation activity in a dosage-dependent manner. (**B**) Time course experiments showed that A2-4-2-4 is required at the early phase of AA-induced AHF. Embryos were treated with AA from 24–72 hpf and A2-4-2-4 added at different times, 24, 30, 36, 42, or 48 hpf. The attenuation of A2-4-2-4 was about 70% when added at 24 hpf but dropped to 35% when added six hours later at 30 hpf (* indicates statistical significance by *t*-test, *p* = 0.038; ** between A2-4-2-4 (36) and A2-4-2-4 (30), *p* = 0.014). A2-4-2-4 lost 90% of the attenuation efficacy when added 24 h later (70% at 24 hpf vs. 7% at 48 hpf). (**C**) A combinational experiment with sub-dosage of the compounds identified in our lab, 10 μM of NS398, 1:500 dilutions of A2-4-2-4, 5 μM of MEK-I, 2 μM of C25, showed a possible synergistic effect between A2-4-2-4 and NS398, comparing the attenuation of double treatment by NS398; A2-4-2-4 (78.6%) with that of lone NS398 (25.2%) or A2-4-2-4 (34.9%) treatment. (* indicates the statistical significance by *t*-test, *p* < 0.05). Similarly, the data suggest a possible synergistic effect between A2-4-2-4 and C25 (* indicates statistical significance by *t*-test, *p* < 0.05 between the double versus single treatments) but an additive effect between A2-4-2-4 and MEK-I.

We tested the dosage effect of A2-4-2-4 by setting up a heart failure experiment with different concentrations. The A2-4-2-4 stock was prepared as described in Section 4.3. The results showed no attenuation by 1:2000 or 1:1000 dilution of A2-4-2-4 but 55%, 70%, and 95% of attenuation by 1:300, 1:200, and 1:100 dilutions, respectively (Figure 4A). We did not test higher concentrations in order to preserve A2-4-2-4 for future experiments. Next, we compared A2-4-2-4 with other drugs identified with our zebrafish model a few years ago [24]. We found that A2-4-2-4 at 1:300 dilution showed approximately the same efficacy of heart failure attenuation as C25 and significantly higher than NS398 and MEK-I. More importantly, unlike C25 and MEK-I, A2-4-2-4 could generate even better attenuation efficacy at higher concentrations with no distinguishable toxicity (Figure 4A, [24]).

Next, we asked when the critical time window of A2-4-2-4 function might be. To test that, we set up time course experiments. Zebrafish embryos were treated with AA at 24 hpf (hours post fertilization) with A2-4-2-4 (1:300 dilution) added at different times, 24, 30, 36, 42, 48 hpf. We let the incubation go to 72 hpf and then the heart failure phenotypes were analyzed. The results showed that when added at 24 hpf, A2-4-2-4 was able to attenuate the heart failure in 70% of the embryos but the attenuation ability significantly dropped when A2-4-2-4 was added six hours later at 30 hpf. The A2-4-2-4 attenuation was almost gone if it was added 12 h later (Figure 4B). These results indicate that A2-4-2-4 functions at the early phase of AHF development.

To study the relationship between A2-4-2-4 and other drugs, we set up combinational treatments, with a sub-dosage of each. In this case, we used 2 μM of C25, 5 μM of MEK-I, and 10 μM of NS398 and A2-4-2-4 (1:500 dilution). The results were very intriguing. Single treatment showed 25.2%, 21.9%, 45%, and 34.9% heart failure attenuation by NS398, MEK-I, C25, and A2-4-2-4, respectively. But the double treatments showed 78.5% by A2-4-2-4; NS398 combined, 62.6% by A2-4-2-4; MEK-I, and 96.7% by A2-4-2-4; C25. These results indicate that A2-4-2-4 might have an additive effect with MEK-I as the double treatment showed approximately the same efficacy (~63%) as the single treatment of each combined (21.9% of MEK-I; 34.9% of A2-4-2-4). A2-4-2-4, however, might have a synergistic effect with NS398 or C25 as the double treatments showed significantly higher efficacy than the addition of single treatments (Figure 4C). In addition, triple or quadruple treatments of these compounds generated no better, sometimes even lower, efficacy, possibly due to toxicity from too many compounds (Figure S2).

### 2.4. A2-4-2-4 Attenuates AHF by Inhibiting Inflammation and Cardiac Hypertrophy

**Figure 5:**
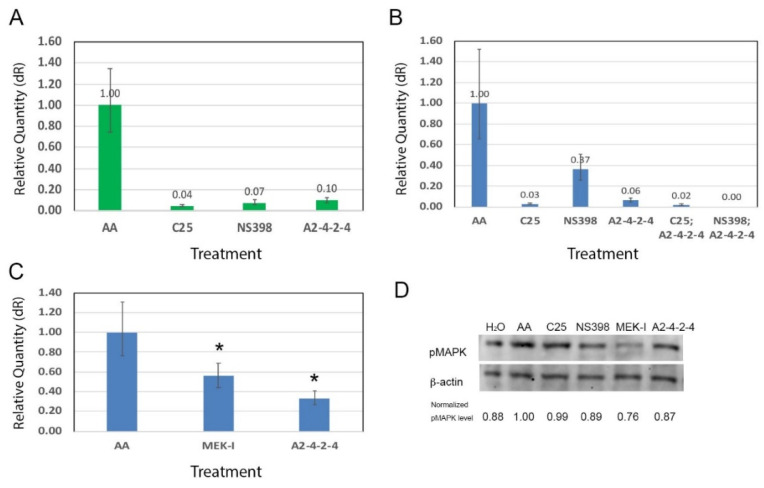
Possible molecular mechanisms of A2-4-2-4. (**A**) The COX-2 gene expression, measured by qRT-PCR, was high in embryos treated with AA alone but significantly lower in those treated with AA plus C25, NS398, or A2-4-2-4. (**B**) A2-4-2-4 showed a synergistic effect with NS398 in suppressing inflammation. While the COX-2 expression level in embryos treated with AA and a sub-dosage of NS398 was 0.37 folds of that in embryos treated with AA alone, the COX-2 level was near zero in the embryos treated with NS398; A2-4-2-4. (**C**) The tnnt-2 expression, an indicator of cardiac hypertrophy, was high in embryos treated with AA alone but significantly lower in embryos treated with AA plus MEK-I or AA plus A2-4-2-4. (* indicates statistical significance between the compound and AA control, *t*-test, *p* < 0.05.) (**D**) Western blotting with anti-phosphorylated MAPK (pMAPK) showed that A2-4-2-4 moderately suppressed the MAPK signaling pathway. The normalized pMAPK level was done by dividing the band intensity in pixels using photoshop of pMAPK by that of β-actin for each sample.

To further understand the molecular mechanisms of A2-4-2-4, we performed RT-qPCR to first test whether A2-4-2-4 would suppress inflammation. We evaluated the inflammation level in the treated embryos by measuring the expression of COX-2 gene. The COX-2 expression level was normalized by that of a housekeeping gene, β-actin, in each sample. As hypothesized, A2-4-2-4 suppressed COX-2 expression as well as NS398 and C25 (Figure 5A). We then wondered if these compounds would have an additive or synergistic effect in suppressing inflammation. To test this, we set up a combinational experiment on zebrafish embryos with a sub-dosage of each compound as before. After analyzing the heart failure phenotypes, the embryos were subjected to RNA extraction, RT, and qRT-PCR. The results showed that when A2-4-2-4 was combined with either NS398 or C25, the inflammation level was lower than single treatment (Figure 5B), which is consistent with the synergistic heart failure attenuation effect of the two drugs observed in Figure 4C.

Since heart failure typically involves cardiac hypertrophy in the early phase of disease development, we wondered whether A2-4-2-4 is involved in cardiac hypertrophy. To test this, we measured the expression of one of the cardiac muscle components, troponin T 2 (tnnt2), by qRT-PCR. The results showed that AA induced a high level of tnnt2 expression but MEK-I moderately suppressed tnnt2 expression (Figure 5C), which is consistent with the known role of the MAPK pathway in promoting cardiac muscle growth [25]. All these results suggest that A2-4-2-4 attenuates AHF by inhibiting both inflammation and cardiac hypertrophy.

### 2.5. A2-4-2-4 and the MAPK Pathway

To test if A2-4-2-4 has any effect on the MAPK pathway, we performed Western blotting to examine the phosphorylated MAPK (pMAPK) level. After obtaining the Western blot images, we measured the intensity of each band in pixels using photoshop and normalized the pMAPK level in each sample using β-actin. After normalization, MEK-I-treated embryos showed a strong reduction of pMAPK, as expected (Figure 5D). A2-4-2-4 actually caused a distinguishably lower level of pMAPK compared with AA alone. NS398 appeared to have no effect on the MAPK pathway, which is also consistent with the non-additive relationship between NS398 and MEK-I in the heart failure attenuation (Figure S2). Interestingly, C25 also caused reduction of pMAPK, as much as A2-4-2-4. This result suggests that even though NS398, C25, and A2-4-2-4 all show an anti-inflammatory function, A2-4-2-4 and C25 have a similar molecular mechanism to NS398.

**Figure 6:**
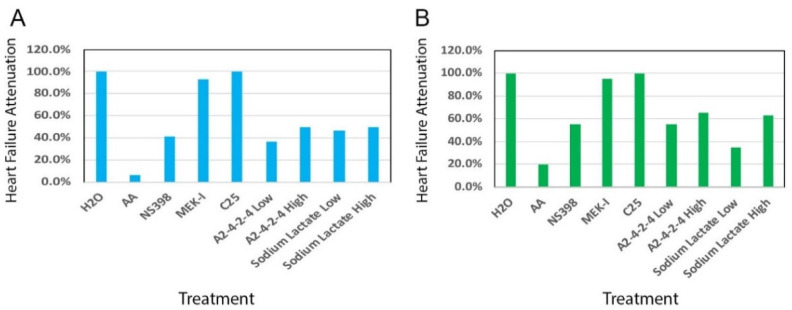
Sodium lactate attenuates AHF. AHF in zebrafish embryos was induced by (**A**) 10 μM AA from 24–72 hpf or (**B**) 5 μM AA from 24–96 hpf, as lower concentration AA takes a longer time to induce severe heart failure phenotypes. Sodium lactate was added to test its AHF attenuation ability at two concentrations (L, 1.5 mM; H, 15 mM). A2-4-2-4 was set up at two concentrations as well (L: 1:1000 dilution lactate; H: 1:300 dilution). Several positive compounds, NS398, MEK-I and C25, were set up for comparison.

To test whether the heart failure attenuation of A2-4-2-4 is actually due to lactate, we set up zebrafish embryos with AA and sodium lactate. With 10 μM AA, the acute heart failure phenotypes typically appeared within 48 h of incubation (Figure 6A, 6.7% attenuation in the AA alone group). The embryos in AA with 15 mM sodium lactate showed 50% attenuation, which was about the same efficacy as A2-4-2-4 and NS398 (Figure 6A). Interestingly, 1.5 mM sodium lactate also showed near 50% attenuation. We then used a lower concentration of AA to induce a less acute heart failure condition in which the severe heart failure phenotypes took a longer time to develop. Sodium lactate showed approximately the same attenuation efficacy (Figure 6B). These results strongly suggest that the AHF attenuation of A2-4-2-4 is attributable to lactate.

**Figure 7:**
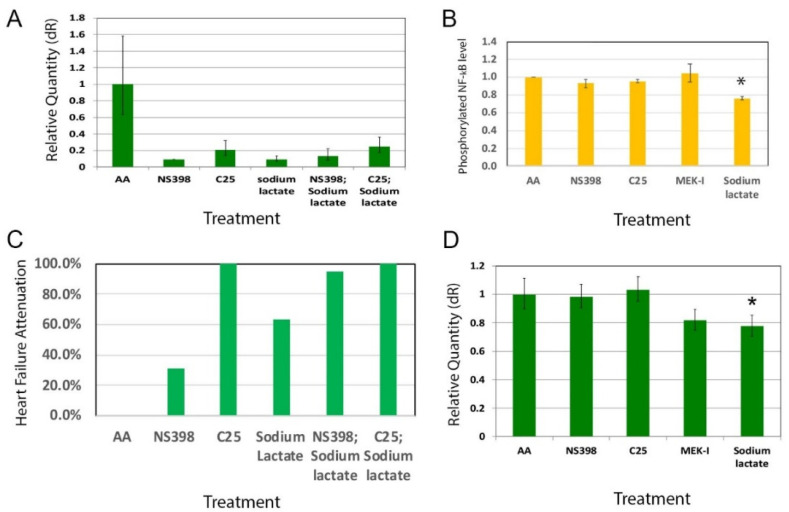
Sodium lactate mechanisms. (**A**) COX-2 gene expression by qRT-PCR in zebrafish embryos treated with AA alone or with test chemicals: NS398, C25, sodium lactate, NS398; sodium lactate, or C25; sodium lactate. Sodium lactate suppresses COX-2 expression. (**B**) Relative levels of phosphorylated NF-κB in zebrafish embryos treated with different compounds. The quantification was done by measuring the pixels of the phosphorylated NF-κB bands and were normalized by that of β-actin on three Western blot images using Photoshop. Sodium lactate treated embryos showed a significantly lower level of phosphorylated NF-κB. (* indicates statistical significance by *t*-test, *p* = 0.001). (**C**) Additive effect of AHF attenuation by NS398 and sodium lactate. (Y axis: heart failure attenuation percentage). (**D**) Tnnt2 expression measured by qRT-PCR was significantly lower in the embryos treated with sodium lactate. (* indicates statistical significance by *t*-test, *p* < 0.05).

To further compare the mechanisms of lactate with A2-4-2-4 in attenuating AHF, we examined the inflammation in sodium lactate-treated AHF fish embryos by qRT-PCR. The results showed that sodium lactate strongly reduced the COX-2 gene expression (Figure 7A). Sodium lactate has been shown to down-regulate the NF-κB signaling pathway [16]. Our Western blot experiments showed that sodium lactate also reduced the level of phosphorylated NF-κB in AHF fish embryos (Figure 7B). Since sodium lactate inhibits inflammation in AHF, we tested whether sodium lactate and NS398 combined would generate a synergistic effect in attenuating AHF. In consistent with the results seen in A2-4-2-4, sodium lactate did show a synergistic effect with NS398 (Figure 7C). Finally, we checked whether sodium lactate also suppresses cardiac hypertrophy as A2-4-2-4 did, and we found that the tnnt2 expression was lower in the sodium lactate-treated AHF embryos (Figure 7D). All these results suggest that lactate is the biological active compound in A2-4-2-4 that attenuates AHF.

## 3. Discussion

### 3.1. Drug Discovery for Acute Heart Failure

Our previous characterizations had shown evidence of cardiac infarction, cardiac hypertrophy and medical response in the AA-induced AHF zebrafish embryos, which are similar to human heart failure, making this model suitable for AHF studies and drug discovery [23]. In the past few years, we have utilized this model to identify several compounds that can attenuate the AA-induced AHF [24]. Among them is NS398, a selective COX-2 inhibitor. A study using a murine chronic heart failure model induced by doxorubicin identified another COX-2 inhibitor to be beneficial to heart failure [26]. The MAPK signaling pathway is known to be involved in inflammation and cardiac hypertrophy during chronic heart failure [27]. We identified MEK-I, an inhibitor of the MAPK pathway, as an AHF attenuator [24]. In this study, we further demonstrated that A2-4-2-4/lactate is an AHF attenuator using our zebrafish AHF model. All these results suggest that the AA-induced AHF model is a great tool to identify compounds that could be beneficial to both chronic and acute heart failure.

### 3.2. Lactate as a Potential Cardiac Protectant

Although A2-4-2-4 is still a mixture of possibly three compounds, with lactate being the major compound, it is very plausible that the heart failure attenuation that we saw by A2-4-2-4 is exerted by lactate. This notion is supported by several lines of evidence. First, Hoque et al., 2014 [16] reported that lactate can reduce the acute injuries in the liver and pancreas caused by the known inflammation-inducing agent lipopolysaccharide. Second, they further showed that lactate protection is through suppressing inflammation. Mice with hepatitis or pancreatitis showed an elevation of many pro-inflammation genes, which was significantly lowered by sodium lactate. A2-4-2-4 attenuates the AA-induced AHF accompanied with a strong suppression of inflammation. Third, since inflammation insult is one of the causes of AHF [7] and an early event in cardiac stress, our results showing that A2-4-2-4 is required in the early phase of AA-induced AHF (Figure 4B) is consistent with our current understanding of AHF. More importantly, we showed that pure sodium lactate can actually attenuate the AA-induced AHF (Figure 7). All these results strongly indicate that lactate could be a cardiac protectant for toxin-induced AHF, perhaps other forms of heart failure as well. Although at this point we are not able to rule out the possibility of the cardiac protection and the molecular mechanisms of A2-4-2-4 being the combined activity of compounds in it, we believe that the possibility should remain low.

### 3.3. Molecular Mechanisms of A2-4-2-4/Lactate

A2-4-2-4 caused a reduction of pMAPK (Figure 5D). Since the MAPK signaling pathway is known to be involved in cardiac hypertrophy [27] and inflammation promotion [12], it is not surprising to see that A2-4-2-4 inhibited inflammation and cardiac hypertrophy (based on the COX-2 and tnnt2 qRT-PCR results, respectively). One of the remaining questions is whether A2-4-2-4 inhibits both inflammation and cardiac hypertrophy through the MAPK pathway singly or together with other pathway(s), independently or sequentially. We hypothesize that A2-4-2-4 is likely to regulate multiple signaling pathways for the following reasons. First, lactate is known to bind GPR81, a Gi-protein-coupled receptor, and inhibit the protein kinase A (PKA)-medicated signaling pathway [16,28]. Lactate was also shown to activate a cAMP/PKA-independent pathway involving β-arrestin to suppress inflammation [16,29]. More interestingly, β-arrestin overexpression has been shown to improve post-myocardial infarction heart failure [30]. Thus, the cardiac protection of A2-4-2-4/lactate is very likely through activating the β-arrestin pathway and inhibiting MAPK and/or PKA pathways. As for inflammation, our results that show an additive relationship between A2-4-2-4/lactate and NS398 suggest that A2-4-2-4/lactate could regulate another inflammation pathway, likely the β-arrestin-mediated pathway. Altogether, it will be interesting to know if and how A2-4-2-4/lactate regulates the Gi-protein coupled receptor-PKA and β-arrestin pathways.

### 3.4. Cellular Mechanisms of A2-4-2-4/Lactate

Inflammation is well known to involve in-wound healing and tissue repair. However, as stated before, prolonged inflammation can cause further tissue damage [13]. It is not surprising to see that inflammation may have a seemingly opposite effect on tissue regeneration involving stem cells. For example, one of the pro-inflammation signaling pathways, NF-κB, was found to maintain the stemness of stem cells [31] but other studies also show that inflammation can induce stem cells to proliferate and differentiate for tissue regeneration [32]. It is getting clear that inflammation levels are the key to determine its constructive or destructive role for tissue regeneration. Since we showed that lactate can attenuate AHF by suppressing inflammation and NF-κB signaling, it is possible that lactate may regulate stem cell behaviors. Interestingly, in a study using C2C12 myoblasts, lactate was shown to promote myoblast differentiation and myotube hypertrophy [33]. The authors further demonstrated that peritoneal injection of lactate in mice can enhance muscle regeneration. All these suggest that lactate may function as a cardiac protectant through regulating stem cell behaviors by fine tuning the inflammation level.

### 3.5. Therapeutic Implication

Our results from the combinational treatments may offer new ideas for AHF treatment. First, A2-4-2-4/lactate showed a synergistic effect with two anti-inflammation agents, NS398 and C25. Although they all inhibit COX-2 expression, the synergistic relationship suggests that multiple inflammation pathways are involved in AHF and could be targeted singly or doubly to produce a better treatment outcome. Alternatively, the synergistic result could be due to the effect of A2-4-2-4/lactate on other pathways, such as cardiac hypertrophy or stem cell differentiation pathways. The additive relationship between A2-4-2-4/lactate and MEK-I yet suggests another potential treatment option for AHF. Since lactate is nontoxic and inexpensive, it has a great potential to become an organ-protective agent. In fact, lactated saline solution has been found to reduce systemic inflammation and produce better outcomes than saline after fluid resuscitation in patients with severe acute pancreatitis [34]. Even though our results received support from other studies, it should be noted that more experiments using a mammalian model and even a human subject are required before the conclusions made by our studies being applied to clinical therapies for AHF in human.

### 3.6. Advantages of Zebrafish Embryo as a Drug Discovery Model

Drug discovery is costly. With accurate disease phenotypes and a properly designed protocol, a zebrafish embryo could be a fast and low-cost animal model for drug discovery [22]. Here we successfully demonstrated the possibility of using a zebrafish embryo to identify new drugs from a natural source that might be beneficial to AHF. The result is exciting in several folds. First, we were able to detect the biological activity of compounds in crude extract, partially purified fraction, and eventually nearly pure forms, likely due to the small size and ready drug accessibility of zebrafish embryos. Second, it leads us to identify lactate, the major compound in A2-4-2-4 fraction, to be a potential cardiac protectant during AHF. Lactate has been shown to be able to protect an injured liver and pancreas [16], but not the heart yet. Third, as we demonstrated that A2-4-2-4 was required during the early phase of AHF and protected the heart by inhibiting inflammation and cardiac hypertrophy, our zebrafish model allowed us to study the pharmacodynamics and molecular mechanisms of A2-4-2-4. In addition, we were able to test the interaction between A2-4-2-4 and other compounds. The zebrafish embryo, as a whole organism system, offers another advantage for drug discovery as it allows us to conduct toxicological tests at the same time. We showed that 15 mM lactate seems to cause no toxicity to the developing embryos.

## 4. Materials and Methods

### 4.1. Chemical Extraction and Isolation

Cooked fruits of *Trapa taiwanensis Nakai* (100 g) purchased from a local market in Taichung City, Taiwan were extracted with H_2_O (C2 extract) or 100% methanol (C3 extract) with ultrasonic vibration three times, 500 mL each. The C3 (9.1 g) crude extract was subjected to a Dianion column (45.0 × 4.0 cm) and eluted with a methanol/water (MeOH/H_2_O) gradient to give rise to five fractions (20%, 40%, 60%, 80%, 100% MeOH). Zebrafish heart failure experiments were performed to identify positive fractions.

The C3-20% fraction was subjected to medium pressure column chromatography (MPLC) with a C_18_ column (15.0 ×1.5 cm column, flow rate: 25 min/mL) again using a MeOH/H_2_O gradient. Ninety-six tubes of eluent were collected and eventually combined based on thin layer chromatography profiles into 10 fractions (named A1~A10). The A1–A10 were dried and redissolved in DMSO at about 100 mM concentration (see chemical preparation in Section 4.3) and tested. Only the A2 fraction exhibited a heart failure attenuation ability (Figure 1B).

The A2 fraction was then chromatographed using MPLC with MeOH/H_2_O (5:95, *v*/*v*) to generate 12 fractions (named A2-1~A2-12), which were tested with the zebrafish heart failure model. The A2-4 turned out to be positive and was separated into 7 fractions (named A2-4-1~A2-4-7) by another MPLC, also with MeOH/H_2_O (5:95, v/v). The A2-4-2 tested positive and was separated into four fractions (named A2-4-2-1~A2-4-2-4) using HPLC and MeOH/H_2_O (5:95, *v*/*v*). The ^1^H, ^13^C, and two-dimensional NMR spectra were obtained using the AVANCE DRX 500 MHz spectrometer (Bruker Instruments, Karlsruhe, Germany) while the ESIMS data were obtained using the MaXis Impact Q-TOF mass spectrometer (Bruker Daltonik, Bremen, Germany).

### 4.2. Zebrafish Husbandry and In Vitro Fertilization

The investigation conforms to the “Guide for the Care and Use of Laboratory Animals” published by the US National Institutes of Health (NIH Publication No. 85-23, revised 1985). The zebrafish stocks used in this study are maintained following standard procedures [35] and are bred by in vitro fertilization. The mature adult male and three female zebrafish were placed in a breeding tank with a divider down the center, separating the two genders. The tanks were then placed in a dark environment until the following morning. The fish were then placed in a concentrated tricaine solution (40 mg/100 mL) to be anesthetized. This process takes about two to three minutes for the fish to be completely anesthetized. Then the females are scooped out and placed briefly on a dry paper towel to dry the body surface. The females are then placed in a 6 cm petri dish to have their eggs expelled. This was done by gently pressing on the lateral and ventral side of the belly using the thumb and index fingers. The eggs were then separated from the female by using a spatula and then gathered into a pile. The female was then transferred back into the breeding tank. After collecting eggs, the male zebrafish was removed, dried, and then placed ventral side up in a cut sponge. Using a dissecting microscope, sperm was collected from the male by placing a capillary tube near the anus while gently pressing on the lateral sides of the belly using tweezers. The sperm was then at once added to the 6-cm petri dish and 1mL of egg water (distilled water containing 60 μg/mL sea salt from Coralife, Franklin, WI, USA) was added to the egg sperm mix. After the eggs are fertilized, the process takes about ten seconds per petri dish, more egg water is added to cover the bottom of the dish. Each petri dish is then labelled and placed in an incubator set at 28.5 °C.

### 4.3. Chemical Preparation and Chemical Treatment on Zebrafish Embryos

In the early stages of the project, as the extracts are a mixture of an unknown number of compounds, we assumed that the averaged molecular weight of these compounds was about 300 g/mole, measured by the weight of dried extracts, and we calculated the volume of DMSO to make 100 mM of extracts. With the assumption that the active compound with biological activity, if any, are likely to make up 10% of the extract, the 100 mM extract should contain 10 mM of the active compound.

Morphologically normal 24-h old embryos were collected and transferred into wells of a 96-well plate, five embryos per well. AA was prepared in egg water with or without herbal extracts or pure compounds, both diluted at 10 μM. The egg water was removed from each well, and 200μL of each chemical treatment was added to each well. The well plate was then paced in the incubator for about forty-eight hours for the chemical treatment to take effect.

At the end of treatment, embryos were dechorionated and analyzed using a dissecting microscope. The AHF phenotypes in our model could be easily classified into four categories: Category I end stage of AHF: heart with no contraction in either cardiac chamber; Category II severe AHF: heart with contraction only in the atrium and no circulation; Category III mild AHF: heart with a distinct contraction in both cardiac chambers but morphological abnormalities, including edema, accumulated blood cells in front of the heart, stretched heart; Category IV normal heart. Video clips of the four categories could be found in Huang et al. [24] Embryos will be tallied into the four AHF categories and the heart failure attenuation is determined by the percentage of categories III and IV combined, i.e., (III + IV)/(I + II + III + IV) %. The control group was embryos incubated with 10 μM AA alone and typically showed near 0% heart failure attenuation at the end of treatment while embryos in water showed 100% attenuation.

Histograms from experiments of the early drug screening phase typically do not contain error bars due to the purpose of these experiments being to identify the positive candidates (Figure 1, Figure 2 and Figure 4A). Statistical analyses were performed for most of the confirmation experiments (Figure 4B and beyond). Error bars indicate the standard deviations of replicates while statistical significance is determined by T-test and is indicated by asterisks on the graphs. Some histograms from later experiments lacking error bars could be due to experiments repeated at different times and/or by different persons and thus only one representative graph is shown.

### 4.4. RNA Extraction

RNA was extracted by placing 20 zebrafish embryos, treated with different compounds, in a tube holding 200 μL of TRIzol. The embryos were then ground until they dissolved and 80 μL of chloroform was added and the tube was inverted ten or more times. The tubes were then centrifuged for ten minutes at 12,500 rpm at 4 °C. The tube now contained two layers, one aqueous and one organic. The aqueous layer was removed and placed in a new tube. Next, 100 μL of isopropanol was added and the tube was inverted to precipitate the RNAs. The tube was left to sit for thirty minutes, after which it was centrifuged at 12,500 rpm at 4 °C for ten minutes. The supernatant was poured off and 100 μL of cold 100% ethanol was added. The tube was then inverted several times, and then spun at 5000 rpm at 4 °C for five minutes. The supernatant was again poured off, and the tube was placed upside down for ten minutes to air dry. The pellet was then resuspended in 10 μL of sterile ddH_2_O and stored at −80 °C.

### 4.5. Quantitative RT-PCR (qRT-PCR)

The extracted zebrafish RNA was used to generate a cDNA library and 5 μg of the total RNA was mixed with double distilled water (ddH_2_O), dNTPs, and oligo(dT)_20_ primer in an Eppendorf tube. The mixture was then placed in a water bath at 65 C for five minutes to denature the RNA. The tube was then removed and placed on ice for a minute, and was then briefly centrifuged. While on ice, 10× RT buffer, 25 mM MgCl_2_, 0.1 M DTT, RNaseOUT (an RNase inhibitor), and Superscript III RT enzyme were added. The tube was then replaced in a water bath at 50 °C for fifty minutes to allow synthesis, then moved to a bath at 85 °C for five minutes to inactivate the enzyme. The cDNA libraries from embryos treated with chemicals were used to set up qRT-PCR tests for the genes β-actin and COX-2, using SYBRgreen and gene-specific primers. The reactions and data analysis were performed on the Mx300P qPCR thermocycler (Agilent Technologies, Santa Clara, CA, USA). The gene specific primers are:

β-actin forward primer: CCTACTAATACACAGCCATGGATGA

β-actin reverse primer: GTCCCATGCCAACCATCAC

COX-2 forward primer: TGTTTTGAACGAGCGGAGTT

COX-2 reverse primer: CAAAGTTGCACATCGATCACA

tnnt-2 forward primer: TTCGGCAAACAGAAATATGAGATCAATGTC

tnnt-2 reverse primer: CACAGCATTCACTTTCTGAGGC [24,36]

### 4.6. Western Blot

Typically, thirty zebrafish embryos treated with chemicals from 24–72 hpf were analyzed under a dissecting microscope for their heart failure phenotypes then transferred to an eppendorf tube. The chemical solution was replaced with 200 μL of lysis buffer (0.2% NP-40, 100 mM Tris, 150 mM NaCl, 8 mM of EDTA, pH 7.4) with a protease inhibitor cocktail (P8340, Sigma, St. Louis, MO, USA) and stored in the freezer. To extract the total proteins, we ground the embryos with pestle then placed the tubes on a rocker in the refrigerator overnight. On the next day, the embryo extracts were centrifuged at 12,000 rpm at 4 °C for 20 min. After the centrifugation, the protein supernatant was transferred to a clean tube and stored in −80 °C. Using the Pierce BCA Assay Kit (23227, Thermo Scientific, Waltham, MA, USA), protein concentrations were analyzed and 10–20 μg of proteins were loaded into a 12% SDS-PAGE gel (BIO-RAD, Hercules, CA, USA) for gel electrophoresis. They were then transferred to 0.4 μm nitrocellulose membrane. Western blotting was performed with the Pierce Fast Western Blot Kit (35050, Thermo Scientific, Waltham, MA, USA). Antibodies were purchased from Cell Signaling Technology.

## Data Availability

Raw data will be shared upon request.

## Supplementary Materials

Supplementary materials can be found at https://www.mdpi.com/1424-8247/14/3/261/s1, Figure S1: AHF attenuation test with crude herbal extracts, Figure S2: Combinational treatment with AHF attenuation compounds.

## Abbreviations

The following abbreviations are used in this manuscript.

AA	aristolochic acid
ADHF	acute decompensated heart failure
AHF	acute heart failure
cAMP	cyclic adenosine monophosphate
COX-2	cyclooxygenase 2
ESIMS	electrospray ionization mass spectrometry
GPR	G-protein receptor
HPLC	high pressure liquid chromatography
JAK-STAT	Janus kinase-signal transducer and activator of transcription pathway
MAPK	mitogen-activated protein kinase
MEK-I	MAPK/ERK kinase inhibitor
MPLC	medium pressure liquid chromatography
NF-κB	Nuclear Factor kappa-light-chain-enhancer of activated B cells
NMR	nuclear magnetic resonance
NSAID	non-steroidal anti-inflammatory drugs
PKA	protein kinase A
qRT-PCR	quantitative reverse transcription-polymerase chain reaction
Tnnt2	Troponin T2, cardiac type
