# Supplementary material for: Identification of Lactate as a Cardiac Protectant by Inhibiting Inflammation and Cardiac Hypertrophy Using a Zebrafish Acute Heart Failure Model
Source: Pharmaceuticals (Basel). 2021 Mar 15;14(3):261. doi: 10.3390/ph14030261 (PMC7999541; doi:10.3390/ph14030261)
Supplement: Supplementary file 1 [file pharmaceuticals-14-00261-s001.pdf]

Supplemental Figures

Figure S1. AHF attenuation test with crude herbal extracts.

Sixty nine crude extracts collected from different parts of about thirty five plants were tested with our AHF zebrafish embryo. Each extract was tested at least four times. Four of them showed consistent attenuation in all four trials (highlighted in yellow). Three of them showed attenuation in 2-3 trials and needed further confirmation (highlighted in blue). Two of them appeared to cause developmental toxicity (highlighted in green). To simplify the chart, the tests that showed zero attenuation are left blank in the AHF attenuation (%) column.

Figure S2. Combinational treatment with AHF attenuation compounds. Zebrafish embryos at 24 hpf were incubated with AA alone or AA with single (blue bars), double (red bars), triple (green bars), or quadruple (blue bar on the right) AHF attenuation compounds. M, MEK-I; NS, NS398; C, C25. Most double treatments showed better efficacy than single treatment whereas triple or quadruple treatments didn't show better efficacy than double treatments.

Supplemental Figure 1

AHF Attenuation Testing with Crude Herbal Extracts

To simplify the chart, the tests that showed no attenuation are left blank in the AHF attenuation (%) column.

No.	Herbal extract	code name	extractive solvent	Used part	AHF Attenuation *(%)*				
					Trial 1	Trial 2	Trial 3	Trial 4	Developmental Toxicity
	Control (AA alone)				40-60		2	5	
1	*Talinum crassifolium*	TC	EtOH		40				
2	*Typhonium divaricatum* (L.) Dence.	TD	EtOH		40				
3	*Kyllinga brevifolia* Rottb.	KB	EtOH		20				
4	*Cuscuta chinensis* Lam	CLE	EtOH						
5	*Pothos chinensis* (Raf.) Merr.	PCR	EtOH	root	40				
6	*Pothos chinensis* (Raf.) Merr.	PCL	EtOH	leave	20				
7	*Pothos chinensis* (Raf.) Merr.	PCS	EtOH	stem					
8	*Duranta repens*	DRF	EtOH	fruit					
9	*Nolina recurvata* (Lem.) Hemsley	1A	EtOH	leave					
10	*Nolina recurvata* (Lem.) Hemsley	2A	EtOH	stem	60				
11	*Yucca elephantipes* Regel	3A	EtOH	leave					Embryos died at 24 hpt [3]
12	*Yucca elephantipes* Regel	4A	EtOH	stem					Embryos died at 24 hpt
13	*Yucca gloriosa* L.	5A	EtOH	leave					Embryos died at 24 hpt
14	*Rohdea japonica*	6A	EtOH	leave	60-80	20			
15	*Rohdea japonica*	7A	EtOH	stem	20				
16	*Dracaena surculosa*	8A	EtOH	leave					

Supplemental Figure 1

17	*Agave potatorum*	9A	EtOH	leave					Embryos died at 24 hpt
18	*Nolina recurvata* (Lem.) Hemsley	1B	EtOH	leave	60				
19	*Nolina recurvata* (Lem.) Hemsley	2B	EtOH	stem	60				
20	*Yucca elephantipes* Regel	3B	EtOH	leave					Tail blister at 24 hpt, died at 48 hpt
21	*Yucca elephantipes* Regel	4B	EtOH	stem					Died at 14 hpt
22	*Yucca gloriosa* L.	5B	EtOH	leave					Embryos died at 24 hpt
23	*Rohdea japonica*	6B	EtOH	leave	40				
24	*Rohdea japonica*	7B	EtOH	stem	20				
25	*Dracaena surculosa*	8B	EtOH	leave					
26	*Agave potatorum*	9B	EtOH	leave	67	20			4/ 6 fish showed rescue
27	*Kalanchoe laetivirens*	YaTing(BuOH[1])	EtOH (BuO1)	whole plant	60	20			
28	*Kalanchoe laetivirens*	YaTing (H_2O)	EtOH (H_2O)	whole plant	80	40			
29	*Kalanchoe laetivirens*	YaTing (EA[2])	EtOH (EA)	whole plant	80-100	60		20	
30	*Kalanchoe laetivirens*	YaTing	EtOH		80	40			
31	*Trapa taiwanensis* Nakai.	a-1	EtOH	cooked pericarp	60				
32	*Trapa taiwanensis* Nakai.	a-2	50% EtOH	cooked pericarp	20				

Supplemental Figure 1

33	*Trapa taiwanensis* Nakai.	a-3	H_2O	cooked pericarp	60				
34	*Trapa taiwanensis* Nakai.	b-1	EtOH	fresh pericarp	60				
35	*Trapa taiwanensis* Nakai.	b-2	50% EtOH	fresh pericarp	40	20			
36	*Trapa taiwanensis* Nakai.	b-3	H_2O	fresh pericarp	80	40	10		
37	*Trapa taiwanensis* Nakai.	c-1	EtOH	cooked fruits	60	20			
38	*Trapa taiwanensis* Nakai.	c-2	50% EtOH	cooked fruits	100	80	60	20	
39	*Trapa taiwanensis* Nakai.	c-3	H_2O	cooked fruits	80	60	20	35	
40	*Trapa taiwanensis* Nakai.	d-1	EtOH	fresh fruits					Died at 32 hpt
41	*Trapa taiwanensis* Nakai.	d-2	50% EtOH	fresh fruits	40				
42	*Trapa taiwanensis* Nakai.	d-3	H_2O	fresh fruits	60				
43	*Anthurium andraeanum* Linden	Chi1	EtOH		20				
44	*Ajuga dictyocarpa* Hayata	Chi3	EtOH						Embryos died at 24 hpt
45	*Salvia plebeian* R. Br.	Chi5	EtOH	whole plant	40				Sick, Less pigment
46	*Portulaca Oleracea* Linn.	Chi8	EtOH		60	20			
47	*Dimocarpus longan*	Chi9 (50%)	50% EtOH	stem	60				
48	*Dimocarpus longan*	Chi10 (95%)	95% EtOH	stem	20	20			
49	*Dimocarpus longan*	Chi12 (50%)	50% EtOH	pericarp	40				

Supplemental Figure 1

50	*Dimocarpus longan*	Chi12 (95%)	95% EtOH	pericarp	80	60	32	20	
51	*Dimocarpus longan*	Chi13	MeOH	leave	60	20			
52	*Ganoderma tsugae*	KGT	EtOH	mushroom	40				
53	*Clerodendrum thomsoniae* Balf.	CCT	EtOH	whole plant	40				
54	*Ruellia tuberosa* Linn.	CRTS	EtOH	stem	80	20			
55	*Dracaena fragrans*	KDFL	EtOH	leave	20	20			
56	*Kalanchoe arborescens*	CKAS	EtOH	stem					
57	*Thymophylla tenuiloba*	CTT	EtOH	whole plant	40				
58	*Ganoderma tsugae*	KGTW	EtOH (H_2O layer)	mushroom	80-100				
59	*Scutellaria rivularis*	CSR	EtOH	whole plant	60	20			
60	*Orthosiphon aristatus* (Blume)Mig.	CoA	EtOH	whole plant	80-100	20			
61	*Litchi chinensis*	CLCB	EtOH	branch (stem)	40				
62	*Ruellia tuberosa* Linn.	CRTL	EtOH	leave	100	40		25	
63	*Paliurus ramosissimus*	KPRW	EtOH (H_2O layer)	whole plant	40				
64	*Ajuga bracteosa* Wall.	CAB	MeOH:CH_2Cl_2== 1:1	whole plant	60				
65	*Ganoderma tsugae*	GTB	*n*-BuOH layer	powder					
66	*Ganoderma tsugae*	KGTB	*n*-BuOH layer	mushroom	80			30	
67	*Oldenlandia corymbosa*	CoC	MeOH:CH_2Cl_2== 1:1	whole plant	40				

Supplemental Figure 1

68	*Ganoderma tsugae*	GTW	EtOH (H_2O layer)	powder	40				
69	*Paliurus ramosissimus*	KPRE (EA layer)	EtOH (EA layer)	whole plant					

1. BuOH, Butanol
2. EA, Ethyl acetate
3. hpt, hours post treatment

The extracts highlighted yellow showed heart failure attenuation consistently in multiple trials, albeit with variations.

The extracts highlighted blue require more tests to confirm the attenuation effect.

The extracts highlighted green did not show heart failure attenuation, instead they showed interesting developmental toxicity.

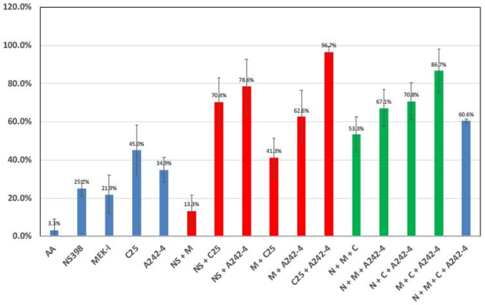

Suppl. Figure 2
